# Comparison of the effect of licorice vaginal cream and estrogen vaginal cream on sexual function of postmenopausal women: An RCT

**DOI:** 10.18502/ijrm.v20i11.12364

**Published:** 2022-12-10

**Authors:** Parisa Ahmadizad, Masoumeh Shohani, Ashraf Direkvand Moghadam, Anahita Jalilian, Hojat Sayadi, Naser Abbasi

**Affiliations:** ^1^Department of Nursing, School of Nursing and Midwifery, Student Research Committee, Ilam University of Medical Sciences, Ilam, Iran.; ^2^Department of Nursing, School of Nursing and Midwifery, Ilam University of Medical Sciences, Ilam, Iran.; ^3^Department of Midwifery, School of Nursing and Midwifery, Ilam University of Medical Sciences, Ilam, Iran.; ^4^Department of Obstetrics and Gynecology, School of Medicine, Ilam University of Medical Sciences, Ilam, Iran.; ^5^Department of Biostatistics, School of Health, Non-Communicable Diseases Research Center, Ilam University of Medical Sciences, Ilam, Iran.; ^6^Department of Pharmacology, School of Medicine, Biotechnology and Medicinal Plants Research Center, Ilam University of Medical Sciences, Ilam, Iran.

**Keywords:** Vagina, Post menopause, Glycyrrhiza, Estrogens.

## Abstract

**Background:**

Menopause is a stage in woman's life that some women experience in middle age and some at a younger age (premature menopause). Low levels of ovarian hormones, during menopause can lead to various complications. Menopause is one of the factors that can affect a woman's sexual function.

**Objective:**

The present study was conducted to compare the effect of licorice vaginal cream and estrogen vaginal cream on the sexual function of postmenopausal women.

**Materials and Methods:**

In this randomized clinical trial study, 82 postmenopausal women who were referred to health centers in Ilam, Iran from July to November 2020 were randomly divided into 2 groups (n = 41/each). One group was given estrogen vaginal cream 2%, and the other vaginal licorice cream 2%. Participants used the 2 medications for 14-day periods each. We used the finite randomization method. Data collection questionnaires, including a demographic information questionnaire before treatment and a female sexual function index questionnaire were completed before, one month after the medication, and 2 months after using the medication.

**Results:**

The mean score of sexual function in the licorice group was 17.86 ± 4.37 and increased to 20.31 ± 4.63 at the end of the study. The mean score of sexual function in the estrogen group was 17.14 ± 3.99 and increased to 22.97 ± 5.09 at the end of the study (p = 0.015).

**Conclusion:**

The effect of estrogen vaginal cream on the sexual function of postmenopausal women was greater than licorice vaginal cream.

## 1. Introduction

Menopause is a stage in woman's life that some women experience in middle age and some at a younger age (premature menopause). This phenomenon is associated with various mental and physical changes and begins in the age range of 45-55 yr. Menopause is a physiological period in a woman's life, the most obvious symptom of which is defined as a permanent interruption of menstruation for 1 yr, which is caused by a decline in estrogen and the destruction of ovarian follicles (1, 2).

Low levels of ovarian hormones, during menopause, can lead to numerous psychological, physical and sexual complications. Some of the symptoms of menopause include vasomotor changes, palpitations, anxiety, insomnia, cardiovascular disease, osteoporosis, changes in the genitourinary system, mood swings, vaginal dryness, and depression. The most common symptoms include night sweats and hot flashes (3, 4). These symptoms may be severe and affect the normal functioning and quality of life (QoL) of postmenopausal women (5).

Sexual dysfunction is defined as a persistent or recurrent decrease in anorgasmia, dyspareunia and sexual arousal (6). Therefore, any disorder that leads to disharmony and as a result, dissatisfaction with sexual intercourse, can lead to sexual dysfunction. Menopause is one of the factors that can affect women's sexual function (7). In postmenopausal women, rugae are lost, and due to reduced vascularity, the appearance of the vagina is almost transparent and pale. Loss of elastic tissue and subcutaneous fat causes labia majora and labia minora to appear wrinkled. In addition, estrogen deficiency, which occurs after menopause, causes atrophic changes and can be associated with symptoms of genitourinary atrophy such as dyspareunia, itching, vaginal burning and vaginismus (involuntary tensing of the vagina) and dryness (6).

The therapeutic measures to improve sexual function during menopause fall into 2 categories; the first category is a hormonal replacement and the second category is the use of alternative and complementary therapies (8). One of the most common hormonal therapies is estrogen, which was used orally in the past. Still, due to its dangerous side effects such as breast cancer and thromboembolism, it is used topically today. Currently available non-hormonal options for treating menopausal sexual disorders include the use of dietary supplements and herbs containing phytoestrogens and isoflavones such as soy, licorice, red clover, and fish oil as well as vaginal gels and lubricants to improve and enhance the QoL in postmenopausal women (9-11).

Due to the high rate of vaginal atrophy, dyspareunia, sexual dissatisfaction, and consequent lack of motivation at this age to seek treatment, postmenopausal women are seriously at risk for poor QoL and mood disorders, including depression. On the other hand, taking hormonal drugs, including estrogen compounds, can expose them to harmful side effects, such as spotting, bleeding, and increased risk of cancer. Therefore, considering this issue and the lower risk of herbal medicines, the researchers decided to compare the effect of these 2 medications on the sexual function of postmenopausal women.

## 2. Materials and Methods

### Study setting

This randomized clinical trial was performed among 82 postmenopausal women from Ilam, Iran in July to November 2020. Using G-Power software and type I error parameters, the test power was 5, the effect size was 87%, the sample size in each group was 34 people and considering the 20% dropout rate, finally 41 people were treated in each group. To perform sampling, the researcher referred to 7 health centers out of 14 health centers in Ilam which randomly selected by lottery method.

### Subjects

Sampling was done based on the following inclusion criteria: 1) at least 1 yr after the onset of menopause and up to 8 yr after the onset of menopause, 2) gynecologist confirmation that shows menopause has occurred, 3) symptoms of vaginal atrophy (dryness, paleness, and dyspareunia), 4) having a spouse, 5) having sex at least twice a month during the research, 6) being able to read or write, 7) body mass index is between 20 and 30 and 8) sexual dysfunction.

The exclusion criteria were: 1) treatment with hormonal drugs, 2) mental illness in the participant or her spouse, 3) presence of underlying diseases such as depression, cancers, pulmonary embolism, diabetes, and 4) taking anticholinergic drugs.

The researcher explained the procedure and the possible side effects to the participants. We used the finite randomization method because we wanted the study groups to have an equal sample size. One of the methods used for finite randomization is the block method. We used the quadratic block method. The 4 blocks consisted of 2 estrogen group participants and 2 participants in the licorice group. And the execution method was that the possible modes for a block of 4 are 6 modes: a) aabb b) abab c) abba d) bbaa e) baba f) baab and to generate random numbers, we used the online kitset software, by first registering the required values from 1-6 (number of digits 6). We repeat this 4 times, but in the 4 ${}^{\text{th}}$ time, the number we put instead of 6 is 2, because in the first 3 times we have produced 18 numbers, and in the last step only 2 more numbers are needed, and thus 20 random numbers between 1 and 6 are produced, which indicate 4 blocks. According to each number, the treatment allocation list is determined. According to the number of samples, 82 people, we formed 20 blocks of 4 and the last 2 people were randomly divided into 2 groups. (A = estrogen group, and b = licorice group), each group included 41 people and demographic characteristics were recorded in the relevant form. Necessary training on how to use vaginal cream, time of sexual intercourse, hygiene about how to use vaginal cream, and time of revisit were provided to the participants by a brochure. Then, group A was provided with estrogen vaginal cream (Stromarine 2% 0.625 mg, manufactured by Abureihan pharmaceutical company, Iran). Group B was provided with vaginal licorice cream 2% (prepared from licorice plant extract, made by the Pharmacological Research Center of Ilam University of Medical Sciences, Iran) along with the applicator. They used the vaginal creams vaginally every night according to the dose determined and approved by the gynecologist (a 5-gr applicator every night). Participants first used the medication for 2 wk and after 2 wk, rested for 10 days, and then used vaginal cream for another 2 wk. The telephone numbers of the participants were obtained, and they were taught not to use any hormonal or other vaginal medication during the experiment. Follow-ups and re-visits from the beginning to the end of the study were done on days 1, 14, 26, 40, and 60.

### Data collection

Data collection questionnaires were completed before the start of treatment (first day of visit), 1 month after use, and 2 months after using vaginal cream with the help of the researcher, and the results were compared. Data collection method included gynecological examinations and a female sexual function index (FSFI) questionnaire. The data collection instruments included a demographic information form and an FSFI questionnaire.

1) Demographic characteristics form that contains information related to age, time of onset of menopause, history of underlying disease, history of medication use, number of previous pregnancies, number of abortions, type of previous deliveries, number of previous deliveries, date of last delivery, substance abuse including smoking and drug abuse, frequency of intercourse per wk, occupation, body mass index, economic status, history of reproductive system surgeries, and education degree.

2) FSFI, a strong and reliable instrument for assessing female sexual function includes 19 questions that address sexual function in 6 domains; desire, lubrication, sexual satisfaction, orgasm, arousal, and pain. A higher score means more sexual satisfaction. The scores of domains are obtained by adding the scores of each domain's questions and multiplying-them by the factor. In the FSFI questionnaire, the number of questions in the domains is not equal to each other, the scores obtained from the questions of each domain were summed and then multiplied by the factor, to balance the domains. Questions were based on 6 subscales of vaginal lubrication (4 items), arousal (4 items), pain (3 items), orgasm (3 items), sexual satisfaction (3 items), and sexual desire (2 items). These subscales have a response range from 0-5. A score of zero indicates that the person has not had sexual activity in the last 4 wk. By summing the scores of the 6 domains together, the total scale's score was obtained. Therefore, a higher score indicated better sexual function. Based on the balancing of the domains, the maximum score was 6 for each domain and 36 for the total scale. The minimum score of sexual desire was 1.2, the domain of satisfaction was 0.8, sexual arousal, vaginal lubrication, orgasm, and the pain was 0, and the minimum score for the total scale was 2 (12).

### Validity and reliability of the instruments

1) Validity of demographic characteristics form by studying similar texts and articles and considering the similarity between their variables and the variables in the present study, this form was prepared by the researcher and presented to 10 faculty members of the School of Nursing and Midwifery and the Medical School of Ilam University of Medical Sciences for approval, and necessary corrections were made.

2) FSFI questionnaire; the reliability and validity of this instrument have been measured in several national and international studies. In the study of Filocamo and colleague, the overall reliability of the instrument showed a Cronbach's alpha coefficents for domain score and total were high, ranging from 0.92-0.97 for the total sample. The test-retest procedure revealed that the concordance correlation coefficient was high both for FSFI-I total score (Pearson's p = 0.93) and for each domain (Pearson's P always > 0.92) of 0.88%, ranging from 0.79% to 0.86% for subscales (12).

Ghassami and colleague achieved the reliability of the Iranian version of this instrument. In 2014. Cronbach's alpha and retest coefficients calculation indicated its favorable end (13).

In the present study, the instrument face and content validity and reliability were assessed again.

### Ethical considerations

This article has been approved by the Ethics Committee of Ilam University of Medical Sciences, Ilam, Iran (Code: IR.MEDILAM.REC.1399.118) and registered in the clinical trial center (last update on April 2022). The participants of the study were assured that their information will be recorded in a completely confidential manner. Written consent was also obtained from the participants of the study to participate in the study.

### Statistical analysis

Data were analyzed after they were entered into satistical package for the social sciences (SPSS), version 22, spss Inc, Chicago, Illinois, USA. Reports were presented as Mean ± SD before and after the intervention. The 3 most common measures of central tendency (mode, mean, and median) and measures of spread (variance, standard deviation, and coefficient of variation) were used. Paired *t* test were used to test the research hypotheses for comparison within the group before and after the intervention. Independent *t* test were used to compare the changes between groups during the study. Repeated-measures analysis was used to evaluate the effect of time in the study. We used analysis of covariance to control possible confounders. The level of significance in all tests was considered to be 5%.

## 3. Results

**Figure 1:**
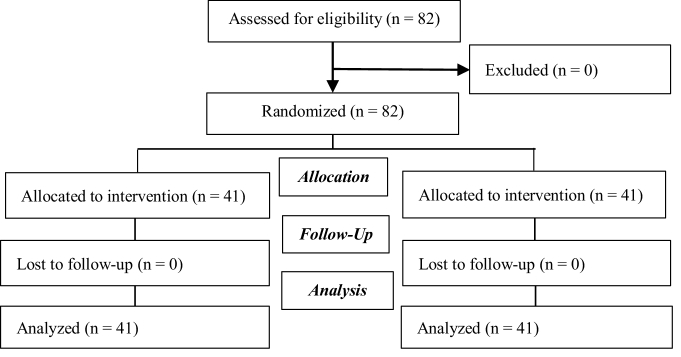
Consort diagram.

The present study was performed on 82 postmenopausal women referred to selected health centers in Ilam. Women who complained of sexual problems and dyspareunia were divided into 2 groups of 41 people (Figure 1).

In the present study no significant difference was observed between the 2 groups in terms of duration of menopause, mean age, age at menarche and other mentioned demographic information (p > 0.05) (Table I).

The results showed that the 2 groups were not significantly different in sexual function before the intervention (p = 0.84). The mean score of sexual desire in estrogen users before the intervention were 2.64 ± 0.869 and in licorice users was 2.23 ± 1.01. The lubrication score was 3.23 ± 0.933 in estrogen users before the intervention and 3.37 ± 1.08 in licorice users. In the 4 ${}^{\text{th}}$ wk, the results showed that the 2 groups were not significantly different in sexual function (p = 0.11). The mean score of sexual desire in estrogen users in the 4 ${}^{\text{th}}$ wk was 3.02 ± 0.930 and in licorice users were 2.47 ± 1.005, which was statistically significant (p = 0.01). The mean lubrication score in estrogen users was 3.66 ± 0.961 and in licorice users was 4.10 ± 0.875, which was statistically significant (p = 0.03). In the 8 ${}^{\text{th}}$ wk, the results showed that the 2 groups were significantly different in terms of sexual function (p = 0.01). The mean score of sexual desire in estrogen users the 8 wk was 3.19 ± 1.08 and in licorice, users were 2.61 ± 0.983, which was statistically significant (p = 0.01). The mean lubrication score in estrogen users at the 8 wk was 4.35 ± 1.006 and in licorice users were 4.009 ± 1.060, which was not statistically significant (p = 0.13) (Table II).

**Table 1:** Demographic characteristics of participants


**Personal characteristic**		**Licorice**	**Estrogen**	**P-value**
Age*		53.8 ± 2.19	53 ± 2.10	0.15
Duration of menopause*		3.56 ± 2	2.97 ± 1.72	0.16
Age at menarche*		13.9 ± 1.37	14.2 ± 1.41	0.27
Sex frequency per month*		3.7 ± 1.45	3.3 ± 1.34	0.17
Gravida*		4.1 ± 1.5	4.6 ± 1.8	0.24
Number of deliveries*		3.7 ± 1.2	4.2 ± 1.6	0.19
Number of abortions*		0.39 ± 0.04	0.34 ± 0.02	0.76
Marriage age*		18.98 ± 2.4	19 ± 2.3	0.96
Body mass index*		26.4 ± 0.44	27.5 ± 0.48	0.05
Education**				
	**Illiterate**	41.52	34	
	**Less than a diploma**	41.48	48.34	0.07
	**Diploma and above**	17	17.66	
Economic situation**				
	**Weak**	7	10	
	**Middle**	51	66	0.06
	**Good**	42	24	
*Data presented as Mean ± SD. **Data presented as percentage. Independent *t* test (Levene's test for equality of variances, *t* test for equality of means)				

**Table 2:** Comparison of changes in sexual dysfunction between groups before the intervention, the 4 ${}^{\text{th}}$ and the 8 ${}^{\text{th}}$ wk after treatment


**Variables**		**Licorice**	**Estrogen**	**P-value**
**Desire**				
	**Before treatment**	2.23 ± 1.01	2.64 ± 0.869	0.84
	**Wk 4**	2.47 ± 1.005	3.02 ± 0.930	0.01
	**Wk 8**	2.61 ± 0.983	3.19 ± 1.08	0.01
**Arousal**				
	**Before treatment**	2.34 ± 0.953	2.75 ± 0.668	0.7
	**Wk 4**	2.49 ± 0.86	3 ± 0.750	≤ 0.001
	**Wk 8**	2.94 ± 0.720	3.16 ± 0.927	0.22
**Lubrication**				
	**Before treatment**	3.37 ± 1.08	3.23 ± 0.0933	0.78
	**Wk 4**	4.10 ± 0.875	3.66 ± 0.961	0.03
	**Wk 8**	4.009 ± 1.060	4.35 ± 1.006	0.13
**Orgasm**				
	**Before treatment**	3.07 ± 1.056	2.83 ± 1.098	0.06
	**Wk 4**	2.92 ± 1.059	3.24 ± 1.25	0.214
	**Wk 8**	2.98 ± 1.24	3.68 ± 1.24	0.01
	**Before treatment**	3.07 ± 1.056	2.83 ± 1.098	0.06
	**Wk 4**	2.92 ± 1.059	3.24 ± 1.25	0.21
	**Wk 8**	2.98 ± 1.24	3.68 ± 1.24	0.01
**Pain**				
	**Before treatment**	3.75 ± 1.050	2.82 ± 0.918	0.70
	**Wk 4**	4.51 ± 0.951	4.80 ± 0.841	0.14
	**Wk 8**	4.88 ± 0.910	4.77 ± 0.796	0.53
**Total**				
	**Before treatment**	17.86 ± 4.37	17.14 ± 3.99	0.84
	**Wk 4**	19.44 ± 4.45	21 ± 4.40	0.11
	**Wk 8**	20.31 ± 4.63	22.97 ± 5.09	0.01
Data presented as Mean ± SD. Independent *t* test (Levene's test for equality of variances, *t* test for equality of means)				

## 4. Discussion

This study aimed to compare the effect of licorice vaginal cream and estrogen vaginal cream on sexual function of postmenopausal women. Considering that the results showed no statistically significant difference between estrogen and licorice groups before the intervention, we can report that the 2 groups were homogeneous in terms of sexual function and individual characteristics.

The present study results showed that the use of licorice vaginal cream had a positive effect on increasing the symptoms of sexual desire, lubrication, satisfaction, orgasm, pain relief and arousal and sexual function increased significantly compared to before the intervention (p = 0.03). The use of estrogen vaginal cream also had a positive effect on increasing the symptoms of orgasm, sexual desire, satisfaction, pain, arousal, and lubrication. It was statistically significant compared to before the intervention (p = 0.02).

At the end of the study (8 ${}^{\text{th}}$ wk), the comparison between the effect of estrogen vaginal cream and vaginal licorice cream on sexual function in postmenopausal women showed that the effect of estrogen vaginal cream on increasing sexual function was greater than licorice vaginal cream and this difference was statistically significant (p = 0.01).

Estrogen improves vaginal mucosa, increases elasticity, increases blood flow into the vulva and vagina, increases tissue thickness, and improves menopausal complications by increasing hyaluronic acid, polysaccharides, and maintaining the functional properties of vaginal epithelial cells (14). Estrogen affects the connective tissue of the reproductive system through 2 types of estrogen receptors, alpha and beta, and the phytoestrogens in plant extracts can bind to estrogen receptors. Since licorice contains phytoestrogenic compounds and the phytoestrogens in this plant act similar to female sex steroid hormones, the possible reason for this action of licorice cream seems to be the presence of estrogen-like compounds in it (14, 15).

The present study demonstrated that the application of licorice vaginal cream can reduce vaginal dryness and subsequently increase vaginal discharge and lubrication, reduce pain, and increase sexual desire. In this regard, our result is consistent with similar study, which was conducted to investigate the effects of jazar supplement (herbal supplement containing Vitex, carrot and fennel seeds) on the QoL and sexual function and vaginal atrophy in postmenopausal women. The study was performed on 90 menopausal women. The participants were divided into 2 groups of 45 intervention and control, the intervention group consumed 4 jazar capsules (500 mg each) and the control group took a placebo for 8 wk. Data was collected using a demographic questionnaire, FSFI, and the Menopause-Specific QoL before the intervention and in week 4, 8, and 10. The results showed that the QoL and mean FSFI score increased significantly compared to the placebo group (p ≤ 0.001), and the study participants had a lower score for vaginal PH and were statistically significant (p ≤ 0.001) (16).

In the present study, vaginal licorice cream, similar to estrogen vaginal cream, significantly reduced dyspareunia in women with sexual dysfunction compared to before the intervention, which is consistent with the results of similar study in which soy isoflavone vaginal gel similar to estrogen vaginal cream improved dyspareunia in postmenopausal women (17).

The present study showed that by applying licorice vaginal cream and increasing sexual function, the QoL in postmenopausal women increases indirectly, which was not in conflict with the results of the study of Asgari and colleagues, to evaluate the effect of licorice on the QoL of postmenopausal women. Here, 380 mg of licorice extract 3 times a day or placebo were used in 2 groups of postmenopausal women. The results showed that the level of QoL was significantly increased in postmenopausal women. In their study, the QoL of 60 postmenopausal women in the 2 groups showed no statistically significant difference before the intervention, but one month after the intervention, a statistically significant difference was observed and shown in the total QoL, psycho-social, vasomotor and physical dimensions and it was shown that using licorice improves the QoL in postmenopausal women (11).

The present study, found that the mean vaginal lubrication in postmenopausal women who used licorice vaginal cream was 3.37 ± 1.08 before the intervention, which reached 4.009 ± 1.060 after the intervention. This difference was statistically significant (p ≤ 0.001). Furthermore, in this study, the mean dyspareunia in women using licorice vaginal cream was 3.75 ± 1.050 before the intervention, which reached 4.88 ± 0.91 after the intervention, which was statistically significant (p ≤ 0.001). The results showed that licorice as a vaginal cream can moisten the vagina and eliminate the unpleasant feeling of vaginal dryness, and decrease dyspareunia and increase the levels of vaginal mucus secretions, which is homogeneous and consistent with others who performed a study to evaluate the effect of licorice vaginal cream on atrophy symptoms among 70 postmenopausal women. In their study, women were divided into 2 groups of 35 and they received 2% licorice vaginal cream and placebo for 8 wk. The results showed that licorice vaginal cream can maintain the normal vaginal flora in postmenopausal women and improve the symptoms and signs of vaginal atrophy (18).

For the time being, non-hormonal options available for the treatment of sexual disorders caused by menopause include the use of dietary supplements and herbs containing phytoestrogens and isoflavones such as soy, licorice, red clover, fish oil, and gels and vaginal lubricants to improve and enhance the QoL in postmenopausal women (11). In the present study, licorice as a phytoestrogen indirectly increased the QoL due to increased sexual function, which was not consistent with the similar study, conducted to investigate the effect of red clover on QoL of postmenopausal women. Fifty-five postmenopausal women participated in their study, of which 27 were in the placebo group and 28 were in the red clover group. At the end of the study, the results showed that the effect of the red clover supplement on the QoL of postmenopausal women was not different from the placebo (10).

In the present study, which was conducted to compare the effect of estrogen vaginal and vaginal licorice cream, the results showed that all 6 domains of the FSFI questionnaire improved after the intervention in the 2 groups of estrogen and licorice, and the difference was statistically significant. The comparison between the 2 groups showed that the increase in orgasm, sexual desire and satisfaction in the estrogen group was greater than in the licorice group. The difference was statistically significant (p = 0.01). The above results were in line with the results of other study, which was conducted to investigate the effect of fennel on the sexual performance of postmenopausal women. In the study, which was performed on 60 postmenopausal women. There were 30 people in the fennel group and 30 people in the placebo group, and each group received treatment for up to 8 wk. Sexual function was assessed using the FSFI, the results showed that in both groups 6 areas of FSFI improved while the difference was more visible in the fennel group (p ≤ 0.001) (19).

Sampling in more centers with higher population diversity, having 2 intervention groups, each of which acts as a control, no drop in samples at any stage of the study, follow-up and visit of patients up to 1 month after the last dose of the drug were the strengths of the study. The time limit of sampling in the field of sexual function studies was the most important limitation of this study due to cultural limitations.

## 5. Conclusion

The use of vaginal licorice cream reduces the symptoms of decreased sexual function (orgasm, sexual desire, lubrication, arousal, pain, sexual satisfaction) in postmenopausal women. In fact, it seems that the maximum effect that vaginal licorice cream has, helps to produce more estrogen in the body or at least maintain its level. The present study showed that the use of vaginal licorice cream over time can increase the score of sexual function symptoms in postmenopausal women and due to less systemic absorption of the drug can be a good option for women with sexual dysfunction. The present study, effect of estrogen vaginal cream on the sexual function of postmenopausal women was greater than licorice vaginal cream. Because the effect of many herbal supplements is determined by long-term use, it is recommended to use licorice for a longer period in the future.

##  Conflict of Interest

The authors declare that there is no conflict of interest regarding the publication of this paper.
